# The use of evidence-based guidance to enable reliable and accurate measurements of the home environment

**DOI:** 10.1177/0308022617737689

**Published:** 2017-11-14

**Authors:** Georgia Spiliotopoulou, Anita Atwal, Anne McIntyre

**Affiliations:** 1 Lecturer in Occupational Therapy, Brunel University London, Uxbridge, UK; 2 Associate Professor, London South Bank University, London, UK; 3 Senior Lecturer in Occupational Therapy, Brunel University London, Uxbridge, UK

**Keywords:** Care Act, equipment abandonment, margin of measurement variation, provision of assistive devices, self-assessment, service user involvement

## Abstract

**Introduction:**

High quality guidance in home strategies is needed to enable older people to measure their home environment and become involved in the provision of assistive devices and to promote consistency among professionals. This study aims to investigate the reliability of such guidance and its ability to promote accuracy of results when measurements are taken by both older people and professionals.

**Method:**

Twenty-five health professionals and 26 older people participated in a within-group design to test the accuracy of measurements taken (that is, person’s popliteal height, baths, toilets, beds, stairs and chairs). Data were analysed with descriptive analysis and the Wilcoxon test. The intra-rater reliability was assessed by correlating measurements taken at two different times with guidance use.

**Results:**

The intra-rater reliability analysis revealed statistical significance (*P* < 0.05) for all measurements except for the bath internal width. The guidance enabled participants to take 90% of measurements that they were not able to complete otherwise, 80.55% of which lay within the acceptable suggested margin of variation. Accuracy was supported by the significant reduction in the standard deviation of the actual measurements and accuracy scores.

**Conclusion:**

This evidence-based guidance can be used in its current format by older people and professionals to facilitate appropriate measurements. Yet, some users might need help from carers or specialists depending on their impairments.

## Introduction

Assistive devices can play an important role in facilitating activities of daily living and social participation in older people with mobility limitations (Cook and Polgar, 2014) by promoting self-efficacy and greater use of adaptive strategies and reducing fear of falling and home hazards (Gitlin et al., 2006). Successful use of assistive devices can also reduce informal carers’ burden by increasing the person’s independence (Mortenson et al., 2013). Despite the reported benefits, currently there seems to be a lack of truly integrating these devices into users’ and their carers’ daily lives resulting in equipment abandonment (Kintsch and DePaula, 2002).

This is a major issue worldwide that has been particularly investigated in the United States (for example, Phillips and Zao, 1993, Riemer-Reiss and Walker, 2000), Australia (for example, Wiedlandt et al., 2006), the Netherlands (for example, DeBoer et al., 2009) and Italy (for example, Federici et al., 2016). The reported abandonment rate varies slightly depending on the methods used to investigate this phenomenon, but also due to the heterogeneity of national health and social care, and private providers’ service delivery systems across countries (Federici et al., 2016). Still, the commonly reported abandonment rate falls within 30% one year after device delivery (for example, Phillips and Zao, 1993; Riemer-Reiss and Walker, 2000). This is a very large percentage associated with huge direct consequences to people’s lack of independence and quality of life, which in a way can be attributed to a failure of the services that have provided the abandoned pieces of equipment (Verza at al., 2006). This also has a cost-related impact for the individual and the national healthcare system (Verza et al., 2006), and it may also result in the loss of economic productivity of informal caregivers associated with the time spent to care for their dependants whose needs cannot be met otherwise (Fast et al., 2001).

Lack of consideration of user opinion in the selection process, poor performance or inappropriateness of the device (Phillips and Zao, 1993), as well as incompatibility of the device with the user’s environment (Ahn et al., 2008) and lack of fit (DeBoer et al., 2009) have been identified as the most influential factors for equipment abandonment.

Federici and Scherer (2012) emphasised the importance of high quality assessment processes in ensuring appropriate fit and matching the user to the device provided.

Assessing for the provision of assistive devices is a complicated process which involves many variables. Orton (2008) suggested that therapists conduct their own risk assessment for the user, the carer and the environment, and they also take into account issues around training and knowledge of the equipment, policies on provision and practice, choices and the ultimate impact of the equipment on care provision. Measuring the relevant furniture in the person’s chosen environment is considered an integral part of the assessment in ensuring fit (Atwal et al., 2017). However, there is extremely limited literature on the exact process of how therapists take accurate measurements, which is one of the most important factors that could impact on fit and matching of the device to the user and the environment. Further to this there is an indication that there might be differences among them in this process resulting in measurement discrepancies (Hoffman and Russell, 2008). Yet, there is lack of evidence to indicate the extent to which these discrepancies would result in the prescription of inappropriate equipment, which may put the person’s functional performance and safety at risk and consequently result in abandonment of the device.

Current practice suggests that therapists would conduct a home visit and take measurements themselves or they may ask users to do so (Isaacson, 2011). The latter could be a powerful way to involve users in the assessment process. Studies have shown that when service users are involved in the issuing process and are properly informed, their satisfaction with these devices and hence their long-term use increases (Martin et al., 2011). However, there is evidence to suggest that therapists question the accuracy and reliability of the measurements taken by users and carers (Money et al., 2011). Spiliotopoulou and Atwal (2014) emphasised the need for providing service users with standardised good quality guidance on how to measure their home environment in order to promote accuracy, ensure the best fit of assistive devices and enable them to participate in the assessment process and even self-assess. Enabling people to self-assess, choose and buy their own assistive devices which would fit appropriately is particularly crucial under the strengths-based approach advocated by the Care Act, 2014, which pushes for collaborative working between service users and practitioners and empowering people to take control and make effective and personalised choices over their care (Social Care Institute for Excellence, 2015).

However, there is currently no standardised guidance which could be used at a national level to inform service users or professionals on the measurement process. Atwal et al. (2017) demonstrated that only 16% of the National Health Service (NHS) trusts and social care services, which participated in a survey aiming to identify guidance that therapists use or provide to service users in enabling them to measure their home environment, did use some sort of guidance. The same authors identified issues with the readability of these leaflets, as well as differences in the suggested ways for measuring furniture. The lack of standardised guidance could consequently lead to inaccurate measurements being submitted by either the professionals involved, especially less experienced staff, or users. Lack of accuracy in measurements may link to lack of fit between the equipment, the person and the environment, and, as mentioned before, to equipment abandonment with financial and quality of life implications. In response to the above issues Spiliotopoulou (2016) developed evidence-based measurement guidance for measuring the person’s popliteal height, baths (length, internal and external width, height), height of the toilet (including and excluding toilet seat), bed height, stairs (length of stairs and two different methods to measure the height of stair rails) and chairs (height, depth, width). The guidance has been made available through the Disabled Living Foundation and Shaw Trust website at http://asksara.dlf.org.uk/brunel, and has been linked to the existing AskSara guided advice about daily living assistive devices to facilitate self-assessment.

This paper reports on the reliability and accuracy testing of this guidance. The current study aims to: (a) investigate the intra-rater reliability of the guidance; (b) investigate whether its use can increase the accuracy of taking measurements; and (c) identify what is considered as acceptable variation in the measurements taken without putting the individual’s performance and safety at risk.

## Method

### Design and process

A within-group design was used to test the accuracy of the developed guidance. As part of this, participants were asked to take measurements of the identified furniture and fittings under two conditions (at time 0 (T0, baseline measurements) without using the guidance and at time 1 (T1) with the guidance) and measurements were compared between the two conditions to see if accuracy was improved. Participants were also asked to take the same measurements twice (T2) with the use of guidance. The intra-rater reliability was assessed to check whether repeated measurements taken by the same person between T1 and T2 obtained similar results.

At T0 participants were provided with a metal tape measure in order to take measurements of specific furniture and record them on the given paper. They were given the option to use either the metric or the imperial system and they had to tick the relevant box on the booklet to indicate their choice. At T1 and T2 participants were asked to measure the same furniture by using a hard copy of the measurement guidance in the format of a booklet. For the popliteal bed and chair height, participants had to measure a standard seated person wearing flat shoes to allow comparisons for the accuracy testing.

After the completion of measurements, participants were also asked to respond in writing to the following question: ‘What do you think is an acceptable margin of error between measurements taken?’. This question was posed further to inform the accuracy testing and enable the researchers to interpret the data obtained, as there was no published information about the acceptable variation in measurements in practice. The session for each participant lasted approximately 2 hours including breaks.

### Participants

The sample included 25 female health professionals (20 occupational therapists, three occupational therapy technicians, one occupational therapy assistant and one senior nurse). Their working experience ranged from 0.5 years to 35 years (mean = 15.9, median = 15, standard deviation (SD) = 9.2) with only two occupational therapists having less than 5 years’ experience. The sample also consisted of 26 older people (13 men and 13 women) between the ages of 60 and 89 years (mean = 69, median = 68, SD = 7.8) who were in need of or have already been using assistive devices. The study took place at the Stoke-on-Trent Mobility and Independent Living Centre. Although the initial aim was to recruit mainly occupational therapists, the occupational therapy technicians, assistant and the senior nurse volunteered to participate as they were also involved in the provision of assistive devices as part of their jobs.

### Procedures

#### Ethics

Ethical approval was granted by Brunel University Research Ethics Committee. Formal R&D permission was also gained from trusts within England through the National Institute for Health Research (NIHR) coordinated system for gaining NHS permission (NIHR CSP), which was accessed via the Integrated Research Application System (IRAS). For recruiting occupational therapists working within social care settings, ethical approval was obtained through a separate centralised system, which was accessed via the Association of Director of Adult Social Services (ADASS). Also all participants provided written informed consent.

#### Recruitment

A national call was issued to target occupational therapists who were involved in the provision of minor assistive devices in any adult NHS and social care setting in England. NHS settings were identified by searching through the online NHS service directories page, and social care settings were identified through the online A–Z list of local councils. In addition, recruitment was facilitated by the Staffordshire and Stoke-on-Trent Partnership NHS Trust. Older people were invited to attend via a newsletter and an advert on the British Polio Fellowship website and health-related user groups. They were also recruited through Stoke-on-Trent Community Health Voice and the local carers’ group.

#### Pilot study

A pilot study was conducted with seven occupational therapists and three service users at the Disabled Living Foundation and Shaw Trust showroom in London. As this resulted in changes to the measurement process, the data from the pilot study were not included in the main analysis.

#### Sample size

The sample size was calculated using the G*power 3 software. For the accuracy analysis, the sample size to ensure a power of 0.80 with a medium effect size of 0.5 (dz) and for a one-tailed hypothesis was 27 participants. For the intra-rater reliability analysis, the total sample size to ensure a power of 0.80 with a medium effect size of 0.5 (ρ) and for a two-tailed hypothesis was 29 participants.

#### Data analysis

All measurements were converted to the metric system by the researchers before the data analysis. The data analysis was carried out using statistical software (IBM SPSS statistics for Windows, v. 20.0). Data were checked for normal distribution by using the Shapiro–Wilk test. Intra-rater reliability was mainly tested with Spearman’s rank test (or with Pearson correlation analysis when the data did meet the parametric assumptions). The alpha level to test for significant correlations was set at 0.05.

Accuracy was measured in terms of discrepancies between participants’ measurements and a ‘criterion set’. This criterion was based on the measurements taken by an expert occupational therapist when using the guidance and was agreed among the research group. Each measurement taken by participants was converted into an accuracy score according to the following formula:

Accuracy score = absolute value (participant’s measurement – criterion set)

As the data were not normally distributed, the Wilcoxon signed rank test was conducted to test for significant differences in the accuracy scores between T0 and T1 and the alpha level was set at 0.05. Descriptive analysis (mean, SD) was also conducted to allow a closer look at the accuracy scores before and after using guidance and provide further insight into the explanation of the inferential statistical analysis. Descriptive analysis was also conducted for the data related to the ‘acceptable margin of variation’.

## Results

### Intra-rater reliability

Pearson’s (*r*) correlation analysis was only used for the popliteal height measurements, which were the only normally distributed data (Shapiro–Wilk test, *P* > 0.05). The Spearman’s rho test was used for the rest of the items. Table 1 presents the results from the intra-rater analysis for the participants as one group and then for each group of participants and for the item-by-item analysis for baths, toilets, stairs, beds and chairs.

**Table 1.** Intra-rater reliability between T1 and T2 when guidance was used.

	Popliteal height	Bath height (AB)	Bath internal width (CD)	Bath total length (EF)	Bath external width (GH)	Toilet height (including seat)	Toilet height (excluding seat)
Group 1	r = 0.70*	rho = 0.38	rho = 0.30	rho = 0.31	rho = 0.47*	rho = 0.56*	rho = 0.87*
	N = 22	N = 24	N = 25	N = 24	N = 25	N = 25	N = 25
Group 2	r = 0.14	rho = 0.32	rho = 0.16	rho = 0.12	rho = 0.52*	rho = 0.10	rho = 0.46
	N = 22	N = 25	N = 25	N = 25	N = 25	N = 24	N = 24
Total	r = 0.42*	rho = 0.35*	rho = 0.26	rho = 0.32*	rho = 0.56*	rho = 0.29*	rho = 0.63*
	N = 44	N = 49	N = 50	N = 49	N = 50	N = 49	N = 49
	Bed height	Stair length	Stair rail (method 1)	Stair rail (method 2)	Chair height (AB)	Chair depth (CD)	Chair width (EF)
Group 1	rho = 0.70*	rho = 0.81*	rho = 0.96*	rho = 1*	rho = 0.18	rho = 0.74*	rho = 0.59*
	N = 24	N = 22	N = 15	N = 5	N = 18	N = 18	N = 18
Group 2	rho = 0.80*	rho = 0.69*	rho = 0.93*	rho = 0.50	rho = 0.56*	rho = 0.91*	rho = 0.74*
	N = 25	N = 24	N = 15	N = 3	N = 23	N = 23	N = 23
Total	rho = 0.82*	rho = 0.73*	rho = 0.93*	rho = 0.90*	rho = 0.37*	rho = 0.78*	rho = 0.641*
	N = 49	N = 46	N = 30	N = 8	N = 41	N = 41	N = 41

*P < 0.05; *N*: number of participants included in the analysis; Group 1: health professionals, Group 2: service users, Total: both groups combined.

Table 1 shows that when the participants were treated as one group, there were statically significant results for all apart from one measurement (bath internal width), for which the *P* value was not far from reaching statistical significance (*P* = 0.072). When the sample was split into one group of health professionals and one group of older people, the results for the health professionals did not identify statistical significance for four measurements (bath height/internal width/length and chair height). The *P* value for bath height was not far from reaching statistical significance (*P* = 0.064). For the older people’s group six out of 14 measurements did not reach statistical significance (popliteal height, bath height/internal length/length, toilet height including seat, and measuring for a stair rail using method 2).

### Accuracy analysis

#### Descriptive analysis: acceptable margin of variation

Fourteen of the occupational therapists responded to this question by providing a specific arithmetic indication for an acceptable margin of error. The reported suggestions ranged from 10 mm to 50.80 mm (mean = 26, SD = 10.21). Twelve older people responded by giving a range between 6.35 mm and 50 mm (mean = 17.93, SD = 13.88).

#### Descriptive analysis: missing data at T0

Some participants (two occupational therapy technicians, three occupational therapists and 13 service users) could not measure specific items without using guidance; hence they did not record these measurements at time T0. In total, two measurements were not recorded by occupational therapy technicians, four measurements by occupational therapists and 34 measurements by older people. Total missing data varied between one and 13 for specific items, with measurement of popliteal height being the most frequently non-recorded without the guidance item. This was mostly evident in the older people’s group (12 out of 26 could not take this measurement without guidance). Table 2 presents the items that were not recorded for each participant at T0. It also indicates which of these items were still not recorded at T1 when guidance was used and indicates the calculated accuracy score for recorded measurements at T1.

**Table 2.** Items that were not measured by participants without guidance (T0), indication of which of these items were not measured when guidance was used (T1) and the accuracy score of measured items at T1.

Participants	Items not measured at T0	Items still not measured at T1	Accuracy score at T1 in mm
OTT 1	Popliteal height	–	–38.60
OTT 2	Bath height	–	104.80
OT 1	Stair length	–	4.00
OT 2	Chair height	–	–2.80
OT 3	Chair depth/toilet height (including seat)	–	73.40/–1.50
SU 1	Bath height	–	104.80
SU 2	Popliteal height/bath height/chair height/chair depth/stair rail height	Popliteal height/stair rail methods 1 or 2	NA/104.80/22.60/–2.80/NA
SU 3	Popliteal height	–	5.00
SU 4	Popliteal height/stair rail height	–	5.00/0.00
SU 5	Popliteal height	–	–0.50
SU 6	Popliteal height	–	–38.60
SU 7	Popliteal height	–	12.20
SU 8	Popliteal height	–	88.40
SU 9	Popliteal height/stair length	–	12.20/4.00
SU 10	Popliteal height/bath length	–	–13.20/14.50
SU 11	Popliteal height/bath height/stair length	–	15.00/101.00/–20.00
SU 12	Popliteal height	Popliteal height	NA
SU 13	All measurements	Stair rail methods 1 or 2	–13.20/104.80/–9.20/1.80/–1.50/ 8.60/11.20/40.60/4.00/22.60/–2.80/–24.00/NA

OT: occupational therapist; OTT: occupational therapy technician; SU: service user; mm: millimetres; NA: non-available as the item was still not measured at T1.

Of the 40 measurements that were not completed without guidance, 36 (90%) were completed when the guidance was used. By looking at the accuracy score calculated for the recorded items at T1, it appears that 29 of the 36 measurements (80.55%) were also within the acceptable margin of variation (up to 50 mm) suggested by participants. Therefore, although inferential statistical analysis between T0 and T1 could not be undertaken (due to lack of measurements at T0) to demonstrate accuracy, the large percentage of recorded measurements that also fell within the suggested margin of variation indicated that to a very large extent, the guidance enabled the participants to take accurate measurements.

#### Descriptive analysis: variation in measurements taken between T0 and T1

The descriptive analysis of the data showed the when guidance was used at T1, the SD and the range for all measurements were considerably decreased. The only SD that did not change significantly was for the chair width (SD 0 = 22.02, SD 1 = 22.01); however, the range at T1 was considerably decreased and also at both times SDs were small in comparison to the mean of these measurements. Table 3 presents the descriptive statistics of the measurements taken at T0 and T1 for the whole group. It was not possible to compare before and after to ascertain accuracy for the height of stair rails, as when users were taking measurements without the guidance, they did not have the two options which were specified afterwards in the guidance as method 1 and method 2.

**Table 3.** Descriptive statistics for measurements taken at T0 and T1 in millimetres (whole group).

Measurements	*N*	Range	Minimum	Maximum	Mean	SD
Popliteal height T0	38	736.60	330.20	1066.80	451.59	110.85
Popliteal height T1	49	183.40	350.00	533.40	433.73	28.18
Bath height T0	46	203.20	381	584.20	550.63	31.27
Bath height T1	50	110.00	450.00	560.00	554.02	17.26
Bath internal width T0	50	905.00	365.00	1270.00	563.01	116.19
Bath internal width T1	51	122.00	508.00	630.00	562.26	16.69
Bath total length T0	49	1600.20	127.00	1727.20	1629.58	277.87
Bath total length T1	50	477.20	1250.00	1727.20	1684.44	68.05
Bath external width T0	50	990.60	558.80	1549.40	712.17	123.92
Bath external width T1	51	203.20	508.00	711.20	697.02	27.71
Toilet height including seat T0	50	457.20	406.40	863.60	480.69	56.92
Toilet height including seat T1	51	32.60	450.00	482.60	474.14	8.65
Toilet height excluding seat T0	49	279.40	419.10	698.50	455.03	37.04
Toilet height excluding seat T1	51	76.20	406.40	482.60	450.35	12.45
Bed height T0	50	663.60	200.00	863.60	461.67	78.12
Bed height T1	51	133.35	400.05	533.40	456.79	36.70
Stair length T0	47	3644.90	254.00	3898.90	1664.31	569.60
Stair length T1	51	2722.20	1151.30	3873.50	1572.94	337.63
Chair height T0	48	762.00	406.40	1168.40	480.46	140.90
Chair height T1	48	292.10	419.10	711.20	459.12	46.24
Chair depth T0	48	838.20	431.80	1270.00	476.42	117.32
Chair depth T1	48	165.10	444.50	609.60	492.40	48.91
Chair width T0	50	170.40	490.00	660.40	529.53	22.02
Chair width T1	48	101.60	457.20	558.80	520.53	22.01

*N*: number of participants; SD: standard deviation.

The descriptive analysis of the accuracy scores, which were calculated by deducting participants’ measurements from a criterion set, also indicated that the SD of the accuracy scores was considerably reduced when guidance was used (see Table 4). Also, the mean of the accuracy scores was always within the acceptable margin of variation as suggested by participants (that is, up to 50 mm). The only measurements that were exceeding this margin were the bath height and the stair length.

**Table 4.** Descriptive statistics of absolute scores in mm at T0 and T1 (whole group).

Measurements	*N*	Range	Minimum	Maximum	Mean	SD
Popliteal height T0	38	621.30	0.50	621.80	47.67	99.99
Popliteal height T1	49	94.50	0.50	95.00	22.24	20.45
Bath height T0	46	127.00	3.20	130.20	99.81	18.45
Bath height T1	50	102.00	4.00	106.00	100.18	16.29
Bath internal width T0	50	702.00	0.00	702.00	41.77	108.38
Bath internal width T1	51	60.00	2.00	62.00	11.58	13.24
Bath length T0	49	1573.00	0.00	1573.00	76.41	276.25
Bath length T1	50	450.00	0.00	450.00	19.64	66.96
Bath external width T0	50	849.40	0.00	849.40	26.84	121.54
Bath external width T1	51	192.00	0.00	192.00	7.85	26.71
Toilet height including seat T0	50	388.60	1.00	389.60	16.04	54.98
Toilet height including seat T1	51	23.00	1.00	24.00	6.57	5.55
Toilet height excluding seat T0	49	251.50	1.00	252.50	12.66	35.94
Toilet height excluding seat T1	51	38.60	1.00	39.60	8.10	10.37
Bed height T0	50	419.10	2.50	421.60	42.25	68.38
Bed height T1	51	89.40	2.00	91.40	30.36	25.10
Stair length T0	47	2398.90	–20.00	2378.90	285.34	512.37
Stair length T1	51	2353.50	0.00	2353.50	76.63	332.96
Chair height T0	48	708.40	0.00	708.40	43.21	135.55
Chair height T1	48	251.20	0.00	251.20	22.09	40.50
Chair depth T0	48	810.00	0.00	810.00	22.61	116.26
Chair depth T1	48	149.60	0.00	149.60	37.98	44.62
Chair width T0	50	127.00	1.40	128.40	10.31	19.56
Chair width T1	48	73.40	1.40	74.80	15.71	19.14

*N*: number of participants; SD: standard deviation.

#### Inferential statistical analysis

The Wilcoxon signed rank test was conducted in order to test for significant differences in the accuracy scores of participants between T0 and T1. The analysis for the health professionals’ group revealed that there were statistically significant differences only for the measurements related to stair length (*P* < 0.001) and chair depth (*P* < 0.042). For the older people’s group, discrepancy reached significance only for stair length (*P* = 0.002), chair depth (*P* < 0.009) and chair width (*P* = 0.022). When both groups were treated as one to increase the power of the test, again the only measurement discrepancies that reached statistical significance were stair length (*P* < 0.001), chair depth (*P* < 0.001) and chair width (*P* = 0.009).

## Discussion and implications

This study demonstrated that a newly developed evidence-based guidance was successful in facilitating health professionals and older people to measure furniture and themselves accurately and reliably . This was also the first study to report on what is considered to be an acceptable margin of variation for measurements taken so that the individual’s functionality and safety are not compromised.

### Reliability of the guidance

The findings support the reliability of the developed guidance, with the bath internal width being the only exemption, which again was close to achieving statistical significance. Bialocerkowski and Bragge (2008) emphasised the importance of a tool to be tested for intra-rater reliability in rehabilitation. Its presence suggests that the effect of differing levels of skills among participants on taking measurements is eliminated (Bartlett and Frost, 2008). The participants of this study were composed of occupational therapists, occupational therapy technicians and one assistant, one nurse and older people. Although the Canadian Association of Occupational Therapists (2006) suggests that all occupational therapists are considered to be experts in the provision of assistive devices, their level of experience could possibly affect the way they take measurements. In this study the average working experience of professionals was 15.9 years, which is quite substantial. However, two occupational therapists had less than 5 years' experience, which might have had a slight impact on the findings; according to Rassafiani et al. (2009) 5 years of experience should be the baseline for recruiting experts for research. Also, the fact that occupational therapy technicians/assistant and the participating nurse did not have the same training in the provision of assistive devices as part of their undergraduate education is another factor that might impact on measurements. Even so, the fact that the intra-rater reliability was supported in this group that also involved older people – who might be considered experts by virtue of their experience (Beresford, 2003), while not having any particular training in taking measurements – pinpoints the success of the guidance to eliminate errors and enable people to measure reliably.

When the group was split into a group of professionals and a group of older people, the results indicated reliability issues with measurements relating to the bath (both groups), the chair height (health professionals), the toilet height, popliteal height and the measurement for a stair rail using method 2 (older people). The main explanation for the lack of statistically significant results for these items relate to the small sample size in the item-by-item analysis for the split groups to allow adequate power of the statistical test in identifying significant correlations (Sterne and Davey Smith, 2001). The suggested sample size for the reliability analysis was 29 participants. The number of participants when the group was treated as a whole ranged from 44 to 50 for the item-by-item analysis, whereas for the split groups it ranged from three to 25. Still, further explanations for these findings have been explored.

For the bath and chair measurements, reliability might have been affected by the diagram used in the relevant bath picture and by the chair picture in the guidance. The bath diagram included four arrows and the sequence of the letters might not have been very intuitive. This was actually supported by the comment of one occupational therapist during the trial of the guidance. Therefore, one may suggest that this aspect along with the fatigue resulting from the repeated measurements might have caused some confusion and hence affected the intra-rater reliability. The importance of including simple pictures and diagrams in leaflets to enhance readability has also been emphasised by other studies (Royal College of Anaesthetists and Association of Anaesthetists of Great Britain and Ireland, 2003). With respect to the chair height, the tested guidance displayed a picture of a standard chair that could not be compressed and did not include directions on considering compressed heights in the measurement process. However, the chair that was provided during the trial could be slightly compressed when a person was sitting on it. Therefore, if the health professionals took measurements once while seated on the chair and once standing up, this would have influenced the reliability of findings. This was a limitation of the study suggesting that further clarifications on measuring compressed heights need to be provided. Indeed, measurement leaflets do make distinctions depending on the type of furniture used, for example type of chairs (Atwal et al., 2017).

The reliability of the popliteal height measurements appeared to be problematic for older people. Popliteal height is the main anthropometric dimension used to determine appropriate seat height (Tuttle, 2004). The anatomical method for determining it was included in the guidance, with the person in an upright sitting position and the popliteal height measured as the vertical distance from the insertion of the biceps femoris tendon to the floor (Pheasant, 1992). Although previous studies (Tuttle, 2004) have suggested that this is a reliable method, it might be challenging for older people and especially for those with mobility issues and associated difficulties in bending. One limitation of this study was the lack of data on the participants’ impairments to determine the extent to which these might have impacted on measurements. However, the challenging aspect of older people measuring the popliteal height themselves has been commented on by participating older people, and it does indicate that some might need help from others (carers, family or professional staff) in taking this measurement.

The reliability of the toilet height measurements was not supported for the older people’s group. This might relate to the design of the leaflet, with the toilet seat pictures blending into the white background and potentially causing confusion for those with mild visual problems. It could also be related to difficulties in bending. As has been mentioned before the lack of data on possible impairments do not allow for a detailed analysis into the above matters. Still, this needs to be explored further by future studies, as the toilet seats have been found to be among the most commonly adopted devices, with their use increasing with age (Cornman et al., 2005). Also, inappropriate toilet height can lead to falls and influence a person’s ability to use the toilet physiologically (Capezuti et al., 2008).

### Accuracy of the guidance

The study aimed to determine whether the guidance could enable people to take accurate measurements and reduce the discrepancies among different parties. Several measures were employed in the data analysis to make the findings more robust (descriptive analysis, inferential statistical analysis, use of a set criterion to compare measurements and use of a reported acceptable margin of variation).

The guidance enabled participants and especially older people to take 90% of measurements that they would not be able to complete otherwise. What is mostly important is that 80.55% of these measurements were also within the acceptable margin of variation as was specified by the participants. This is a very promising result as it indicates that the guidance can enable users to take accurate measurements themselves. Hence, it can allow them to exercise control and make decisions about the provision of assistive devices and achieve personal goals, which is actually very important under the strengths-based approach advocated by the Care Act, 2014 (Social Care Institute for Excellence, 2015). It could also allow professionals to trust the measurements taken by service users as this has been a point of caution in previous research (Money et al., 2011).

The descriptive analysis clearly demonstrated that the guidance improved accuracy and significantly reduced variation across participants for each one of the measurements taken. This was clearly demonstrated by the reduction of the SD of the actual measurements and the accuracy scores, which were calculated by comparing participants’ measurements to a criterion set. Hence, the results support the use of the guidance in increasing consistency and accuracy in measurements for health professionals and older people. Atwal et al. (2017) emphasised the importance of confirming measurement techniques in occupational therapy and by doing so reducing potential measurement errors. Such guidance could be used in the education of trusted assessors, occupational therapy assistants and occupational therapy students to inform evidence-based teaching. It could also be used among occupational therapy graduates to eliminate any discrepancies in measurements resulting from the use of different practices (Hoffmann and Russell, 2008). The above findings are very important as inaccurate measurements may impact on the suitability of the devices to match the person’s environment and hence result in safety issues, equipment abandonment and a reduction in quality of life for people with disabilities, as they would be unable to complete their occupational roles and everyday life activities for which the use of a device could be helpful (Ahn et al., 2008; DeBoer et al., 2009).

However, the non-parametric statistical analysis could not identify statistically significant changes in the accuracy scores for most items (apart from stair length, chair depth and chair width) before and after the use of the guidance. Apparently, this was due to the low power of the statistical test to detect such small differences for most measurements, as the initial calculation of the sample size was based on a medium effect size of 0.5 (dz). Also, the sample size calculation was based on the use of parametric statistical analysis, which could not be performed due to the lack of normally distributed data. Hence, a significantly larger sample would be needed for the current analysis in comparison with the original estimate (Kanyongo et al., 2007).

### Acceptable margin of variation

There is no previous research to establish the effect of measurement error on assistive device users or what margin of error is considered acceptable (Atwal et al., 2017). This was the first study that attempted to gather information on the acceptable margin of variation, which was determined by both older people and health professionals to a maximum of 50 mm. This finding was also used as a guide in identifying to what extent the use of guidance allowed for ‘acceptable inaccuracies’ in comparison to the criterion set. The findings suggested that for most measurements the mean discrepancy was smaller than the suggested upper limit, apart from the bath height and the stair length; however, the mean discrepancy even for the stair length was considerably decreased when guidance was used and even achieved statistical significance during the comparison analysis. Yet, one needs to be cautious when using this upper limit in determining the accuracy of measurements. This study had a considerably small sample size of 26 participants responding to this item, which makes the results difficult to generalise. Also, the study was limited in asking participants to suggest the acceptable margin of measurement variation without relating this to specific furniture measurements.

Still, the fact that there was agreement between older people and health professionals in that matter is an interesting finding per se, as previous research that had compared therapists’ perception of best fit for chairs with that of an older adult had identified a significant difference between the expert’s and the client’s score (Odunaiya et al., 2014). These findings are also a reminder that assistive device users are aware of the importance of measurements impacting on safety and function, and can be considered as trusted advisors and even experts in the assessment process for the provision of assistive devices.

## Conclusion

The results from the current study were very positive and suggest that the developed guidance can be used in its current format by older people and specialists in enabling them to take accurate and reliable measurements. Its use by specialists could promote a more timely, safe, consistent and transparent way of assessment and fitting of assistive devices. Older people can use the guidance to measure for the provision of simple assistive devices; however, they might still need some help from carers or specialists (for example, with measuring their popliteal height) depending on the severity of their disability. Although this guidance is a way to make the assessment process more inclusive for the service users in terms of taking measurements, it does not consist of a decision-making tool for the type of equipment. However, these findings highlight the possibility of more inclusive approaches to assistive devices provision and set the stage for econometric studies assessing cost-effectiveness.

Future research needs to look at the applicability of this guidance both with service users and with professionals involved in the provision of assistive devices. Future studies could look at how this guidance could be applied in other countries taking into account cultural and anthropometric differences that might occur. Although different delivery systems for assistive devices provision exist in different countries (Federici et al., 2016), there is definitely a need to provide guidance for the use of professionals and service users on the best measurement practices in order to increase safety and independence and minimise the cost related to equipment abandonment. Finally, larger studies need to explore in more depth the acceptable margin of variation in measurements taken for different types of equipment taking into account the variety of users’ needs.

## Key findings

- This guidance can enable service users and health professionals to measure home furniture reliably and accurately.
- Service users should be encouraged to use this guidance either independently or with assistance.

## What the study has added

This study provided an insight into how measurement guidance could be used to ensure the best fit of equipment, as well as initial information on the acceptable variation in measurements taken.

## References

[bibr1-0308022617737689] Ahn M Beamish JO Goss RC (2008) Understanding older adults’ attitudes and adoption of residential technologies. Family and Consumers Sciences 36(3): 243–260.

[bibr2-0308022617737689] Atwal A McIntyre A Spiliotopoulou G et al.(2017) How are service users instructed to measure home furniture for provision of minor assistive devices? Disability and Rehabilitation: Assistive Technology 12(2): 153–159. 27376636 10.3109/17483107.2015.1111942

[bibr3-0308022617737689] Bartlett JW Frost C (2008) Reliability, repeatability and reproducibility: analysis of measurement errors in continuous variables. Ultrasound in Obstetrics and Gynecology 31(4): 466–475. 18306169 10.1002/uog.5256

[bibr4-0308022617737689] Beresford P (2003) It’s our lives: a short theory of knowledge, distance and experience, London: Citizen Press.

[bibr5-0308022617737689] Bialocerkowski AE Bragge P (2008) Measurement error and reliability testing: application to rehabilitation. International Journal of Therapy and Rehabilitation 15(10): 422–427.

[bibr6-0308022617737689] Canadian Association of Occupational Therapists(2006) CAOT position statement: assistive technology and occupational therapy. Available at: http://www.caot.ca/default.asp?pageid = 598. 12704975

[bibr7-0308022617737689] Capezuti E Wagner L Brush BL et al.(2008) Bed and toilet height as potential environmental risk factors. Clinical Nursing Research 17: 50–66. ##. 18184978 10.1177/1054773807311408

[bibr8-0308022617737689] Cook AM Polgar JM (2014) Assistive Technologies: Principles and Practice, 4th ed St.Louis: Mosby.

[bibr9-0308022617737689] Cornman JC Freedman VA Agree EM (2005) Measurement of assistive device use: Implications for estimates of device use and disability in later life. Gerontologist 45(3): 347–358. 15933275 10.1093/geront/45.3.347

[bibr10-0308022617737689] DeBoer IG Peeters AJ Ronday HK et al.(2009) Assistive devices: usage in patients with rheumatoid arthritis. Clinical Rheumatology 28: 119–128. 18726549 10.1007/s10067-008-0989-7

[bibr11-0308022617737689] Fast J Eales J Keating N (2001) Economic impact of health, income security and labour policies on informal caregivers of frail seniors, Ottawa: Status of Women Canada Available at: http://www.familycaregiversbc.ca/wp-content/uploads/2015/04/informal-caregivers-in-canada-a-snapshot-2001.pdf (accessed 15 October 2016).

[bibr12-0308022617737689] Federici S Scherer MJ (2012) Assistive Technology Assessment Handbook, Boca Raton, FL: CRC Press.

[bibr13-0308022617737689] Federici S Meloni F Borsci S (2016) The abandonment of assistive technology in Italy: a survey of National Health Service users. European Journal of Physical and Rehabilitation Medicine 52(4): 516–526. 26784731

[bibr14-0308022617737689] Gitlin LN Winter L Dennis MP et al.(2006) A randomised trial of a multicomponent home intervention to reduce functional difficulties in older adults. Journal of the American Geriatric Society 54(5): 809–816. 10.1111/j.1532-5415.2006.00703.x 16696748

[bibr15-0308022617737689] Hoffmann T Russell T (2008) Pre-admission orthopaedic occupational therapy home visits conducted using the internet. Journal of Telemedicine and Telecare 14: 83–87. 18348754 10.1258/jtt.2007.070808

[bibr16-0308022617737689] Isaacson M (2011) Best practices by occupational and physical therapists performing seating and mobility evaluations. Assistive Technology 23(1): 13–21.

[bibr17-0308022617737689] Kanyongo GY Brook GP Kyei-Blankson L et al.(2007) Reliability and statistical power: how measurement fallibility affects power and required sample sizes for several parametric and nonparametric statistics. Journal of Modern Applied Statistical Methods 6(1): 81–90.

[bibr18-0308022617737689] Kintsch A DePaula R (2002) A framework for the adoption of assistive technology. Paper presented at SWAAAC 2002: Supporting Learning Through Assistive Technology, *Winter Park, CO, USA Available at: http://l3d.cs.colorado.edu/clever/assets/pdf/ak-swaaac02.pdf (accessed 20 October 2016)*.

[bibr19-0308022617737689] Martin JK Martin LG Stumbo NJ et al.(2011) The impact of consumer involvement in service delivery on satisfaction with and use of assistive technology. Disability and Rehabilitation 6(3): 225–242. 20929425 10.3109/17483107.2010.522685

[bibr20-0308022617737689] Money A McIntyre A Atwal A et al.(2011) Universal access in human–computer interaction: applications and services. In: Stephanidis C (ed.) Universal Access in Human-Computer Interaction. Applications and Services, Orlando, FL: Springer, pp. 4556.

[bibr21-0308022617737689] Mortenson WB Demers L Fuhrer MJ et al.(2013) Effects of an assistive technology intervention on older adults with disabilities and their informal carers. An exploratory randomized controlled trial. American Journal of Physical Medicine and Rehabilitation 92(4): 297–306. 23291602 10.1097/PHM.0b013e31827d65bf PMC5484629

[bibr22-0308022617737689] Odunaiya NA Owonouwa DD Oguntibeuju (2014) Ergonomic suitability of educational furniture and possible health implications in a university setting. Advances in Medical Education and Practice 5: 1–4. 24511247 10.2147/AMEP.S38336 PMC3915023

[bibr23-0308022617737689] Orton M (2008) Factors that may be considered by occupational therapists during the assessment of clients for assistive technology and whether it permeates through to the eventual prescription. Journal of Assistive Technologies 2(1): 11–22.

[bibr24-0308022617737689] Pheasant S (1992) Bodyspace: Anthropometry, Ergonomics and Design, London: Taylor & Francis.

[bibr25-0308022617737689] Phillips B Zhao H (1993) Predictors of assistive technology abandonment. Assistive Technology 5: 36–45. 10171664 10.1080/10400435.1993.10132205

[bibr26-0308022617737689] Rassafiani M Ziviani J Rodger S et al.(2009) Identification of occupational therapy clinical expertise: decision-making characteristics. Australian Occupational Therapy Journal 56(3): 156–166. 20854509 10.1111/j.1440-1630.2007.00718.x

[bibr27-0308022617737689] Riemer-Reiss ML Walker R (2000) Factors associated with assistive technology discontinuance among individuals with disabilities. Journal of Rehabilitation 66(3): 44–50.

[bibr28-0308022617737689] Royal College of Anaesthetists (RCA) and the Association of Anaesthetists of Great Britain and Ireland (AAGBI) (2003) Raising the Standard: Information for patients, The Royal College of Anaesthetists.

[bibr29-0308022617737689] Social Care Institute for Excellence(2015) Care Act, 2014: Strengths-based approaches. Available at: https://www.scie.org.uk/care-act-2014/assessment-and-eligibility/strengths-based-approach (accessed 18 September 2017).

[bibr30-0308022617737689] Spiliotopoulou G(2016) Summary of key findings of UKTROF funded project on “National guidance for measuring home furniture and fittings to enable service user self-assessment and successful fit of minor assistive devices”. Available at:https://www.rcot.co.uk/sites/default/files/UKOTRF-Key-findings-GS-Brunel.pdf (accessed 20 July 2017).

[bibr31-0308022617737689] Spiliotopoulou G Atwal A (2014) Embedding the personalization agenda in service users’ self-assessment for provision of assistive devices. British Journal of Occupational Therapy 77(10): 483.

[bibr32-0308022617737689] Sterne JA Davey Smith G (2001) Sifting the evidence – what’s wrong with significance tests? BMJ 322: 226–231. 11159626 10.1136/bmj.322.7280.226 PMC1119478

[bibr33-0308022617737689] Tuttle NA (2004) Comparison of methods used for measuring popliteal height. Ergonomics Australia 18(1): 14–18.

[bibr34-0308022617737689] Verza R Carvalho Lopes ML Battaglia MA et al.(2006) An interdisciplinary approach to evaluating the need for assistive technology reduces equipment abandonment. Multiple Sclerosis 12(1): 88–93. 16459724 10.1191/1352458506ms1233oa

[bibr35-0308022617737689] Wiedlandt T McKenna K Tooth L et al.(2006) Factors that predict the post-discharge use of recommended assistive technology (AT). Disability and Rehabilitation: Assistive Technology 1(1–2): 29–40. 19256165 10.1080/09638280500167159

